# Morphological, Thermal, and Mechanical Properties of Nanocomposites Based on Bio-Polyamide and Feather Keratin–Halloysite Nanohybrid

**DOI:** 10.3390/polym16142003

**Published:** 2024-07-12

**Authors:** George Mihail Teodorescu, Zina Vuluga, Andreea Ioniță, Cristian Andi Nicolae, Marius Ghiurea, Augusta Raluca Gabor, Valentin Rădițoiu, Monica Raduly, Ioana Andreea Brezeştean, Daniel Marconi, Ioan Turcu

**Affiliations:** 1 National Institute for Research & Development in Chemistry and Petrochemistry—ICECHIM, 202 Spl. Independentei, 060021 Bucharest, Romania; george.teodorescu@icechim.ro (G.M.T.); andreea.afilipoaei@icechim.ro (A.I.); cristian.nicolae@icechim.ro (C.A.N.); marius.ghiurea@icechim.ro (M.G.); raluca.gabor@icechim.ro (A.R.G.); vraditoiu@icechim.ro (V.R.); monica.raduly@icechim.ro (M.R.); 2 Department of Molecular and Biomolecular Physics, National Institute for Research and Development of Isotopic and Molecular Technologies, 67-103 Donath, 400296 Cluj-Napoca, Romania; ioana.brezestean@itim-cj.ro (I.A.B.); daniel.marconi@itim-cj.ro (D.M.); ioan.turcu@itim-cj.ro (I.T.)

**Keywords:** chicken feather keratin, bio-polyamide, halloysite, nanohybrid, nanocomposite properties

## Abstract

One solution to comply with the strict regulations of the European Commission and reduce the environmental footprint of composites is the use of composite materials based on bio-polymers and fillers from natural resources. The aim of our work was to obtain and analyze the properties of bio-polymer nanocomposites based on bio-PA (PA) and feather keratin–halloysite nanohybrid. Keratin (KC) was mixed with halloysite (H) as such or with the treated surface under dynamic conditions, resulting in two nanohybrids: KCHM and KCHE. The homogenization of PA with the two nanohybrids was conducted using the extrusion processing process. Two types of nanocomposites, PA–KCHM and PA–KCHE, with 5 wt.% KC and 1 wt.% H were obtained. The properties were analyzed using SEM, XRD, FTIR, RAMAN, TGA, DSC, tensile/impact tests, DMA, and nanomechanical tests. The best results were obtained for PA–KCHE due to the stronger interaction between the components and the uniform dispersion of the nanohybrid in the PA matrix. Improvements in the modulus of elasticity and of the surface hardness by approx. 75% and 30%, respectively, and the resistance to scratch were obtained. These results are promising and constitute a possible alternative to synthetic polymer composites for the automotive industry.

## 1. Introduction

The drastic directives imposed by the EU to reduce the use of fossil fuels, to increase interest in the use of renewable resources (the EU target to reach net zero emissions by 2050) [1], and to improve the lifetime of polymer composites, recovery, reuse and recycling of materials (e.g., the EU has a target from 2015 regarding the obligation to reuse and recycle 85% of the vehiclesat the end of their life cycles) [2,3] have led to an increase in the interest of manufacturers in the automotive industry in the use of bio-based polymer composite materials reinforced with natural fibers. There are premium car manufacturers that have invested for some time in high-performance polymer composites reinforced with natural fibers and have already launched vehicles with interior components made of these composite materials. The value of the production of polymer composites based on natural fibers is estimated to reach USD 16.5 billion by 2030, with an annual growth rate of 7% during the forecast period [4]. Although natural fibers (e.g., flax, hemp, jute, and kenaf) are ecological, light, resistant, and low-cost and can replace glass fibers and mineral fillers in many applications in the automotive sector, their use is particularly limited by water absorption and weak adhesion at the interface with the polymer matrix.

To meet the specific requirements of the automotive field, composite materials based on high-performance polymers are used in the manufacture of components. Polyamides (PAs) are widely used to obtain injected parts due to their special properties (e.g., chemical resistance, and mechanical performance) and ease of processing [5]. Considering, on the one hand, the high demand for lighter PA products with improved performance and, on the other hand, the reduction of fossil fuel reserves, scientists have been forced to research and develop PA from sustainable alternative sources.

PA1010 is a semi-crystalline polymer produced 100% from natural renewable resources. Due to the long aliphatic segments and the high CH_2_/CO-NH ratio, PA1010 absorbs a small amount of water, melts at a low temperature (approx. 200 °C), and shows high flexibility, which is why it is recommended for use in the packaging industry [6,7]. At the same time, being a non-biodegradable polymer, it is used in fields that require durable materials, such as in the automotive industry for fuel tubing [8]. However, PA1010 has inferior properties to synthetic polymers in terms of stiffness, mechanical strength, and scratch resistance, and for some auto parts manufacturers (e.g., gear and bearing), it is necessary to improve these properties [8]. A commonly used method for improving the properties of PAs is to use silicate or silica nanoparticles. Thus, Baniasadi et al. [9] succeeded in obtaining, using in situ polymerization, PA614 with 2% organophilized nanoclay and improved the tensile strength and modulus of elasticity by 25% and 30%, respectively. Yan and Yang [10] obtained an increase in the storage modulus by approx. 80% for PA1010 with 1.2 wt.% silica nanospheres with amine groups on the surface, prepared by in situ melt polycondensation.

Although partially or totally bio-based PAs are already commercially available and have good mechanical properties and high chemical and/or high thermal stability, the synthetic grades PA6 and 6.6 are still widely used because they are two to five times cheaper. A way to reduce the cost of bio-PAs and to maintain their quality as “green materials” is to replace some of the bio-polyamide with bio-based fillers while maintaining or even improving its properties. Many scientists have turned to the use of natural materials such as chicken feathers, which are waste products generated by the poultry industry. Feathers are an interesting material with a “self-assembled and multifunctional structure” consisting mainly of calamus, rachis, barbs, and barbules. Almost half of the composition of chicken feathers is feather fibers (barbs), and the other half is represented by rachis with a tubular structure, both containing over 90% keratin, an insoluble protein with high resistance to biodegradation. Two different structures have been identified inside the keratin fibers: a primary structure of microfibrils twisted into an ordered and crystalline structure (α-helix), responsible for the high mechanical strength of the fibers, and a secondary structure of protofibrils inside the microfibrils, the β-sheet structure, randomly twisted [11]. Chicken feather fibers have low density, good thermal stability up to 220–225 °C [12], hydrophobic behavior, with a water contact angle greater than 90°, which suggests a good interface with polymers [13], and very good mechanical properties (i.e., a tensile strength of about 200 MPa, elongation at break of about 7%, and Young’s modulus of about 4000 MPa), comparable to those for cotton fibers and that of coconut fibers [14]. In addition, they are low-cost, biodegradable, non-abrasive, and a continuously renewable keratin resource. Due to these properties, feather keratin could be a promising reinforcing agent in high-performance polymers, contributing at the same time to the reduction of natural waste and to its rational utilization. The efficiency of keratin as a reinforcing agent for different synthetic polymer matrices (polyethylene (PE), polypropylene (PP), polymethyl methacrylate (PMMA) and epoxy resin) [13,15,16,17] or for biodegradable polymers (Polylactic acid (PLA) and Polyhydroxyalkanoates (PHAs)) [18,19,20] has been evaluated by various researchers. In these polymer matrices, different amounts (1–50 wt.%) of keratin fibers with different lengths and thicknesses or in powder form, such as barbs, barbules, rachis, quill (powder), or whole feathers, have been incorporated. For PMMA, an increase in the Young Modulus of about 30% was noticed for 5 wt.% of fibers. The thermal stability of this composite also increased, proving the interaction between polypeptide chains from keratin and PMMA (i.e., the Tg of PMMA increased in the composite from 72 °C to 109 °C, and the maximum rate of decomposition moved to a temperature that was higher by about 30 °C). The results obtained for the composites based on PE revealed a dependence between the polymer-fibers interface and the PE crystallinity (low crystallinity, strong interface). In PP composites, 35 wt.% keratin fibers decreased the acoustic and mechanical properties compared to those obtained with jute fibers. The alkali treatment of feathers allowed an increase in the strength and stiffness of composites compared to untreated feathers. With whole chicken feathers, PP composites presented better flexural strength than composites reinforced with feather fibers (barbs) and higher tensile strength and tensile modulus than composites reinforced with powder of chicken feather quill. The HDPE/PP composites based on chicken feather fibers or quills showed superior sound absorption than composites based on jute. By combining feather fibers with cellulose fibers (kraft pulp fibers), the properties of PP composites increased, especially the mechanical strength. Using maleinized PP, large improvements in strength and modulus (with about 30%) were obtained. PLA and keratin fiber composites had decreased tensile strength, but the elastic modulus was 16% higher than PLA and the keratin fiber composites had the best thermal stability, for 5 wt.% of keratin fibers, while the elongation at break had a maximum value at 2 wt.% of keratin fibers (an increase of 56%). The biodegradability of PLA increased with an increase in the amount of keratin feather fibers. Composites based on PHAs and 1% keratin presented good mechanical properties and had the potential to be used in fully renewable packaging applications. However, to the best of our knowledge, there are no data in the literature regarding the use of keratin materials as reinforcing agents in bio-PA.

The direct use of keratin materials after simple physical processing (i.e., separation, cutting, washing, drying, and grinding) as reinforcing agents in polymer materials is a relatively simple method. However, scaling up and obtaining homogeneous composite materials with improved and reproducible properties is difficult to achieve. That is why, recently, researchers have focused on the regeneration of keratin from natural keratin materials using different extraction methods (e.g., using chemical, thermal, enzymatic hydrolysis, or dissolution in ionic liquids methods or combinations thereof). Through rigorous control of the extraction conditions, regenerated keratin with excellent mechanical properties and in different forms (e.g., nanoparticles, films, fibers, and gel) can be obtained [21]. Regenerated keratin has found many applications, especially in cosmetics and biomedicine, where it is being used either individually or in admixture with other natural or synthetic polymers. Depending on the application, solutions, hydrogels, films, fibers, spongy structures, patches, etc. were obtained [11]. However, to our knowledge, there are no reported studies on the use of regenerated keratin as a reinforcing agent in obtaining polymer composites by melt homogenization under dynamic conditions.

The aim of our work was to obtain new nanocomposites based on bio-PA1010 and hydrolyzed keratin, a commercial product extracted from chicken feathers by chemical hydrolysis in combination with enzymatic treatment. To increase the thermal resistance of both keratin and bio-PA, a special nanosilicate (halloysite nanotubes) was used, which was mixed with keratin under dynamic conditions to obtain a nanohybrid, further used as a bio-PA reinforcement agent. Based on our previous research [22], to improve the dispersion of halloysite in the PA matrix and obtain nanocomposites with improved thermal and mechanical properties, the surface of the silicate nanotube was modified with ethylene bis stearamide. To our knowledge, this is the first time such a nanocomposite has been developed. The morphological, thermal, and mechanical properties of the newly developed bio-PA nanocomposites were analyzed using SEM, XRD, FTIR, RAMAN, TGA, DSC, tensile/impact tests, DMA, and nanomechanical tests to appreciate their utility for automotive applications.

## 2. Materials and Methods

### 2.1. Materials

NP BioPA1010-201, Polyamide 1010 100% bio-based (PA), with high mechanical resistance, good chemical stability, and low moisture absorption, designed for injection applications, was supplied by NaturePlast (Ifs, France) and was used as a polymer matrix. DuPont™ Fusabond^®^ N493, an anhydride-modified ethylene copolymer, with a density of 0.87 g/cm^3^ and MFR (190 °C/2.16 kg) of 1.6 g/10 min, was supplied by DuPont Company (Wilmington, DE, USA) and was used as compatibilizing agent. The commercial hydrolyzed keratin, extracted from chicken feathers by chemical hydrolysis in combination with enzymatic treatment (KC), characterized by protein ≥ 90.0%, moisture ≤ 5.0%, ash ≤ 7.0%, pH 4.8–7.5, and average molecular weight < 2000, was produced by Xi’an D-Sung Health Biotehnological Technology Co., Ltd. (Xi’an, China). Halloysite (H), silicate nanotubes (HNT), with a kaolin content > 95% and a quartz content < 1%, was produced by ABC Company (Rochester, NY, USA). *N*,*N*′-Ethylenebis (stearamide) (E), having a melting range of 144–146 °C, a molecular weight of 593.02 g/mol, and a density of 0.97 g/cm^3^, was supplied by Sigma Aldrich (Saint Louis, MO, USA) and was used as a dispersion agent.

### 2.2. Preparation of Nanohybrids

The nanohybrids were obtained under dynamic conditions in a Brabender Plastograph (GmbH & Co KG, Duisburg, Germany) at 80 °C and 100 rpm for 1 h by mixing KC either with H in a 5:1 ratio or with HE (halloysite with the surface previously treated with 30% dispersing agent E, under dynamic conditions, at 120 °C and 100 rpm for 1 h, as described in our previous article [22]), in a ratio of 5:1.43. The two prepared nanohybrids, labeled, KCHM and KCHE, respectively, were further used as reinforcing agents to obtain PA nanocomposites.

### 2.3. Preparation of Bio-Polyamide Nanocomposites

The PA, previously dried for 2 h at 100 °C, was mixed with 2.5% compatibilizing agent and with 6% KCHM or 6.43% KCHE in a rotary gravimetric mixer for 10–15 min, at room temperature. The resulting mixture was homogenized in the melt in a double-screw extruder, equisens, LSM 30.34 Leistritz (Leistritz Extrusionstechnik GmbH, Nürnberg, Germany), at a rotation speed of the main screws of 80–100 rpm and a temperature on the extruder head of 200–215 °C. The resulting yarns were picked up by a conveyor belt, cooled with air, and then granulated in a granulator mounted in flow with the extruder, resulting in nanocomposite granules marked with PA–KCHM and PA–KCHE, respectively. The resulting granules were dried for 2 h at a temperature of 100 °C, after which standard injected specimens were obtained for physical–mechanical characterization using the injection molding machine Engel Victory VC 60/28 TECH (Engel, Schwertberg, Austria). The test specimens were obtained using the following parameters: the temperature profile of 180–200 °C, the injection pressure of 900 bar, and the injection speed of 0.2 cm^3^/s.

### 2.4. Characterization

#### 2.4.1. Scanning Electron Microscopy (SEM)

The morphological characteristics of the polymer composites were analyzed using a Hitachi TM4000 Plus microscope (Hitachi, Tokyo, Japan) with an accelerating voltage of 15 kV. The tensile test specimen’s active zone was isolated by cutting it and immersing it in liquid nitrogen prior to the SEM investigation. After one minute, the samples were removed from liquid nitrogen, and the specimen’s selected zone was broken up into approximately equal-sized pieces using pliers. A Q150R Plus (Quorum Technologies, SXE, Lewes, UK) equipment was used to coat the fragments with a 5 nm gold layer.

#### 2.4.2. X-ray Diffraction Analysis (XRD)

The Rigaku Smartlab diffractometer (Rigaku Corporation, Tokyo, Japan) was utilized to gather X-ray diffraction data using CuKα1 radiation (λ = 1.5406 Å). During the tests, the acceleration voltage of the generator radiation was set to 45 kV, and the emission current was maintained at 200 mA. Diffractograms at room temperature were gathered using parallel beam geometry, scanning at 2θ = 2° to 60° in 0.02° continuous increments on a scanning pace of 4°/min. Bragg’s equation was applied to identify the interplanar distance (d) of (polymer) in composites. The error margin was kept at ±0.05%. Crystallite sizes, heights, intensity values, and FWHM (full width at half-maximum intensity of the diffraction peak) were obtained using Rigaku PDXL 2 data analysis software. The Powder Diffraction File^TM^ (PDF) from the International Centre for Diffraction Data (ICDD) was used to identify crystalline phases. Diffraction profiles were fitted with curves to isolate individual diffraction maxima.

#### 2.4.3. Fourier-Transform Infrared Spectroscopy (FTIR)

The FTIR spectra were obtained using a JASCO 6300 FTIR spectrophotometer (JASCO Int. Co., Ltd., Tokyo, Japan), equipped with a Golden Gate ATR (crystal of diamond) from Specac Ltd. (London, UK), featuring a spectral resolution of 0.2 cm^−1^ and 0.1% T accuracy. Data were recorded in transmission mode (30 scans) over a range of 4000–400 cm^−1^.

#### 2.4.4. Raman Spectroscopy

A miniaturized BW-TEK i-Raman^®^ Plus Portable Raman Spectrometer (B&W TEK, Newark, DE, USA) with a 785 nm laser line, a maximum power of 350 mW, and a detector of high quantum efficiency CCD arrays, equipped with an optical microscope and objectives of 20×, 50× and 100×. For the specific measurements, an objective of 20× was employed, and the following parameters were set: 10% laser power, 20 s acquisition time, and 1 scan per point. All the spectra were processed with Origin 7 software, performing background subtraction and normalization of the spectra to the most intense peak.

#### 2.4.5. Thermal Characterization

A TA-Q5000IR (TA Instruments, New Castle, DE, USA) thermogravimetric analyzer was used for thermogravimetric analysis (TGA). Nitrogen was used as a purge gas, with a flow rate of 40 mL/min. Samples that weighed 7–8 mg were heated at a rate of 10 °C/min from 25 °C to 700 °C.

A differential scanning calorimeter (DSC; Q2000, TA Instruments, New Castle, DE, USA) was employed to analyze the sample’s melting and crystallization properties: melting temperature (T_m_), normalized enthalpy of melting (ΔH_m_), crystallization temperature (T_c_), and normalized enthalpy of crystallization (ΔH_c_). In a single run, the heat–cool–heat (HCH) approach was used. The process started with a 2 min equilibration at −75 °C. Afterward, the sample was heated to 240 °C, held there for two minutes, cooled to −75 °C, held there for another two minutes, and then heated again to 240 °C at a rate of 10 °C/min. The analysis was conducted under 5.0 grade helium (99.999%) with a flow rate of 25 mL/min. The degree of crystallinity, $X_{C}$, was calculated based on the following formula:

$$X_{C}(\%)=\frac{∆H_{m}}{(1-\emptyset )∆H_{m}^{o}}\times 100\%$$

where $∆H_{m}$ and $∆H_{m}^{o}$ represent the heats (J/g) of melting of PA nanocomposites and of 100% crystalline PA with a value of 244 J/g [10], respectively. ∅ represents the weight fraction of the components incorporated in the PA matrix.

#### 2.4.6. Mechanical Properties Analysis

In accordance with ISO 527 [23], the samples’ tensile properties were assessed using the Instron 3382 universal testing machine (Instron Corporation, Norwood, MA, USA). Tensile strength and modulus of elasticity were determined at 50 mm/min and 1 mm/min, respectively, for seven specimens in each test. The impact strength of the samples was determined using a Zwick HIT5.5 Pendulum Impact Tester (Zwick Roell AG, Ulm, Germany), with seven specimens per test, and by applying the Charpy notched impact test in accordance with ISO 179-1/1 e A [24].

#### 2.4.7. Dynamic Mechanical Analysis (DMA)

The dynamic mechanical properties, storage modulus (E′), loss modulus (E″), and loss factor (tan δ) of nanocomposites as a function of temperature were measured with DMA Q800 (TA Instruments, New Castle, DE, USA) using the temperature ramp method. Samples with rectangular geometry of 60 × 10 × 4 mm (length × width × thickness), cut from injected specimens, for dual cantilever clamp, were scanned with a heating rate of 3 °C/min, from room temperature to 150 °C, at a frequency of 1 Hz in air and an amplitude of 20 µm. The resulting plots were obtained with the Universal analysis V4.5A program.

#### 2.4.8. Nanomechanical Properties Analysis

A TI Premier system (Hysitron Inc., Minneapolis, MN, USA) was used to test nanoindentation and nanoscratching properties utilizing a three-sided pyramidal Berkovich tip (radius of curvature of 150 nm and total angles of 142.35 deg). Samples were placed on metal plates and thoroughly cleaned with 96% ethanol to get rid of any impurities and dust from the surface of the samples. To gather precise and comprehensive data, each sample underwent 20 indentations at a constant force. The values of hardness (H) and reduced modulus (Er) were determined by applying a trapezoidal load function (5 s loading, 2 s hold, 5 s unloading) and recording load-displacement curves at a force of 10,000 µN. The load-displacement plots are generated automatically using TriboScan software version 9. By performing nanoscratching using a constant load scratch of 5000 µN only load function, the coefficient of friction (µ = LF, µN/NF, µN) was determined, and representative 25 µm in situ SPM images were obtained.

## 3. Results and Discussion

### 3.1. Characterization of Feather Keratin–Halloysite Nanohybrids

#### 3.1.1. SEM Analysis

**Figure 1:**
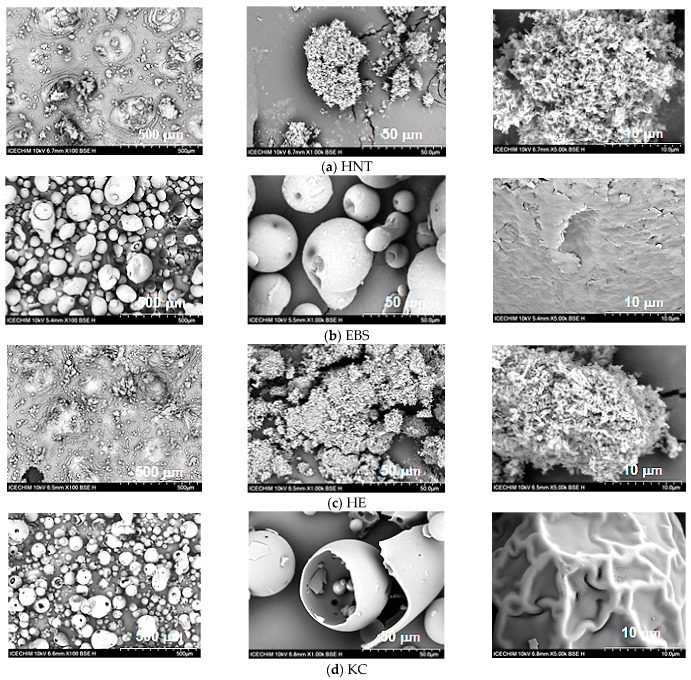

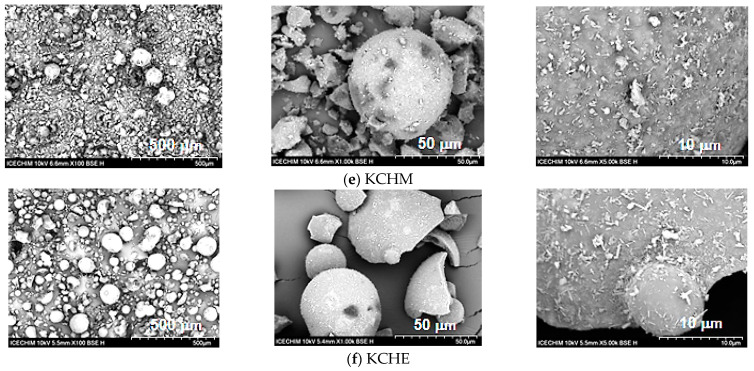
SEM surface morphology at ×100, ×1000 and ×5000 of (**a**) HNT; (**b**) EBS; (**c**) HE; (**d**) KC; (**e**) KCHM; and (f) KCHE.

The surface morphologies of KCHM and KCHE nanohybrids compared to EBS, HNT, and HE are presented in Figure 1. Figure 1a shows SEM images of HNT powder. HNT particles can be observed, mainly in the form of submicron agglomerates of nanotubes and very few isolated nanotubes. EBS particles are small, almost spherical, with dimensions of 5–6 µm or large elongated, with dimensions of 150–200 µm and with an uneven surface. (Figure 1b). By mixing it under dynamic conditions with HNT, EBS was uniformly dispersed on the surface of silicate nanotubes (HE sample), which were maintained as submicron agglomerates of nanotubes (Figure 1c). From Figure 1d, it can be observed the keratin particles in an almost spherical shape of 5–200 µm diameter or in pieces of broken spheres, with holes and a porous internal structure inside which smaller particles are located. The hollow spheres have either a smooth outer surface with a wall thickness of 3–5 µm or a wrinkled surface as an irregular network of chains. By mixing it under dynamic conditions with HNT, it can be observed (Figure 1e) that most of the spherical keratin particles are crushed in the form of pieces of different sizes whose surface is covered with HNT in the form of agglomerates of nanotubes with sizes from 0.1 µm to 1.5 µm (KCHM nanohybrid). Furthermore, some embedding of HNT particles on the surface of keratin particles can be observed, which is evidence of the existence of an interaction. When keratin is mixed with HNT treated on the surface with EBS (KCHE nanohybrid), it is observed (Figure 1f) that the keratin particles mostly retain their spherical shape, and the percentage of crushed particles after mixing under dynamic conditions is lower. At the same time, it is observed that HE uniformly covers the surface of the keratin particles, and the percentage of agglomerated silicate nanotubes is greatly reduced. This behavior is due to EBS, which has the property of reducing friction and abrasion.

#### 3.1.2. X-ray Diffraction Analysis

**Figure 2:**
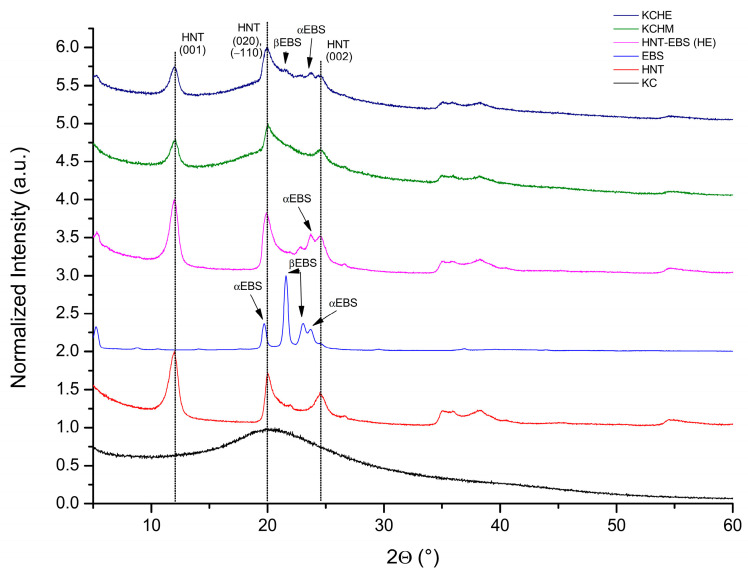
X-ray diffraction patterns of KCHM and KCHE nanohybrids compared to KC, HNT, EBS, and HE.

Figure 2 shows the X-ray diffractograms of KCHM and KCHE nanohybrids compared to keratin, HNT, EBS, and HE. Keratin, KC, shows a broad peak of high intensity at 2θ = 20.2°, characteristic of the folded β-sheet structure [11]. The characteristic peaks for HNT are at 2θ = 11.97°, 19.96°, and 24.54°, corresponding to the (001), (020)/(−110), and (002) planes (ICDD PDF Card 01-081-9524 (halloysite) [25]). The diffractogram of EBS shows peaks at 2θ of 19.69° and 23.72°, characteristic for α crystallographic form, and peaks at 2θ of 21.57° and 23.05°, characteristic for β crystallographic form [26]. In the case of HE, it is observed that the peak corresponding to HNT at 2θ = 19.96° shifts slightly to smaller angles (19.85°), and the width and intensity of the peak increase by approx. 32%, respectively 52%, and the crystallite size decreases by approx. 30%. This behavior is evidence of an interaction between EBS in alpha crystalline form and HNT. The corresponding α EBS peak at 2θ = 19.69° is no longer found in HE, and the one at 2θ = 23.72° decreases a lot in intensity (by approx. 900%). In the case of nanohybrids, the peaks corresponding to HNT widen by 30–53% and decrease in intensity by 30–83%, proof of a disorder in the structure as a result of the interaction between HNT and keratin. The peak change percentage is higher in the case of the KCHM nanohybrid, which is proof of a stronger interaction at the keratin–HNT interface.

#### 3.1.3. FTIR Analysis

**Figure 3:**
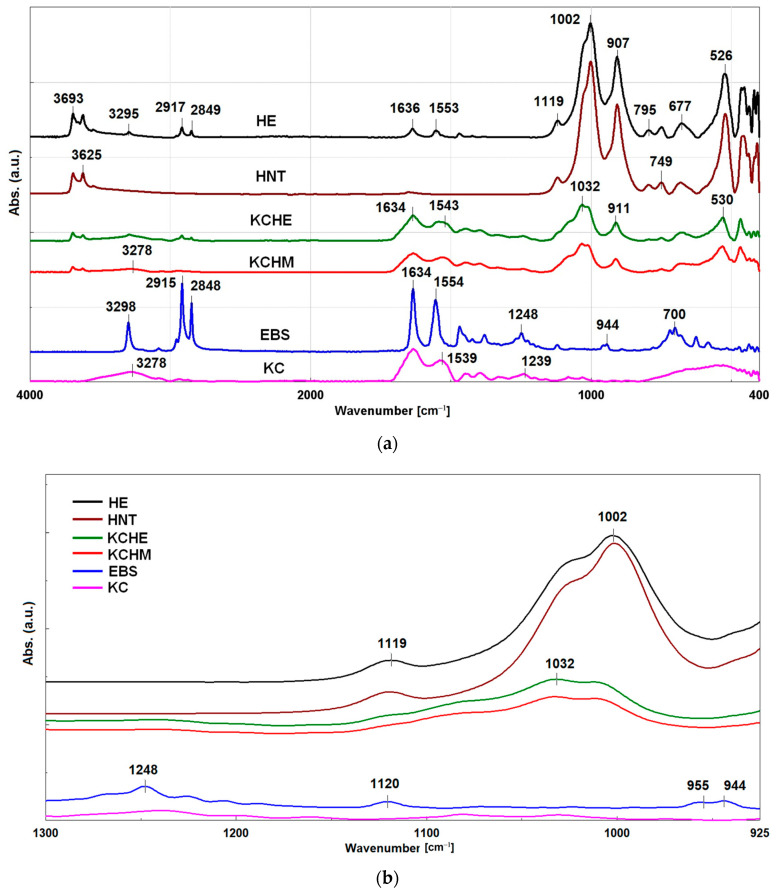
FTIR spectra of (**a**) KCHM and KCHE nanohybrids compared to KC, HNT, EBS, and HE; (**b**) detail with the bands between 1300–925 cm^−1^.

In Figure 3a, the FTIR spectra of of KCHM and KCHE nanohybrids compared to KC, HNT, EBS, and HE are presented. There are characteristic absorption bands for –CO–NH peptide bonds of KC at 1633 cm^−1^, 1539 cm^−1^ and 1239 cm^−1^, which resulted from the stretching vibrations of the C=O bond in Amide I, from the in-plane bending vibrations of the N-H bond in Amide II and from the stretching vibrations of the C-N and C-H bonds and the bending of the N-H bond in Amide III. The absorption band at 3278 cm^−1^ resulting from the stretching vibration of N-H and OH bonds is typical of the Amide A [27,28]. The bands of Amide I and II can also be observed in EBS at 1634 cm^−1^ and 1554 cm^−1^. In Figure 3b, the bands characteristic for α and β crystalline forms of EBS can be observed at 1248 cm^−1^, 957 cm^−1^, and 944 cm^−1^ [29]. After thermodynamical mixing it with HNT (HE sample), the peak at 1248 cm^−1^ disappeared, and the other two peaks (957 cm^−1^ and 944 cm^−1^) are difficult to identify due to the overlap with the characteristic peaks for HNT. Even only the disappearance of the peak at 1248 is evidence of an interaction between HNT and EBS in the alpha form [22]. Furthermore, the peaks of EBS, corresponding to the CH_2_ asymmetric and symmetric stretching vibrations at 2915 and 2848 cm^−1^ and the absorption band for N-H stretching vibration at 3298 cm^−1^ [30,31], are observed, while in the case of HE, the corresponding peaks are located at 2917, 2849 and 3295 cm^−1^, respectively. The band at 3625 cm^−1^, characteristic of HNT, is attributed to the –OH stretching vibration of the structural hydroxyl groups (near the aluminum atoms). This band is found in HE, but with lower intensity (Figure 3a), proving the interaction with EBS through the hydrogen bond between the aluminol (Al-OH) groups and the amide groups. In KCHM and KCHE hybrids, the band at 1634 cm^−1^ specific for Amide I and the band at 1239 cm^−1^ specific for Amide III can be observed with low intensity, especially in the case of KCHM. At the same time, the band at 1539 cm^−1^, characteristic of Amide II shifted a lot to lower wavenumbers in the case of KCHM and very little to higher wavenumbers in the case of KCHE, probably due to the overlap of the peaks at 1539 cm^−1^ of KC and at 1554 cm^−1^ of EBS. The corresponding band for Amide A, at 3278 cm^−1^, is observed only in KCHM. In KCHE, it probably overlaps with the 3295 cm^−1^ peak of HE. In the two hybrids, KCHM and KCHE, the characteristic bands of HNT are found [9,32,33,34]. Compared to HNT, in the hybrids, we can see bands with decreased intensity at about 3624 cm^−1^, corresponding to the vibration of the OH group, and at 911 cm^−1^, corresponding to the bending vibration of Al-OH (slightly blue-shifted compared to 906 cm^−1^ in HNT; Figure 3a). At the same time, we can observe the disappearance of the band at 1119 cm^−1^, corresponding to the Si-O stretching mode, and the increase in the intensity of the band at 1033 cm^−1^ and 1032 cm^−1^, corresponding to the Si-O-Si stretching vibration (Figure 3b). This behavior confirms the interaction between keratin and HNT and the formation of hydrogen bonds between the amide groups and the aluminol and/or silanol groups.

#### 3.1.4. Raman Analysis

**Figure 4:**
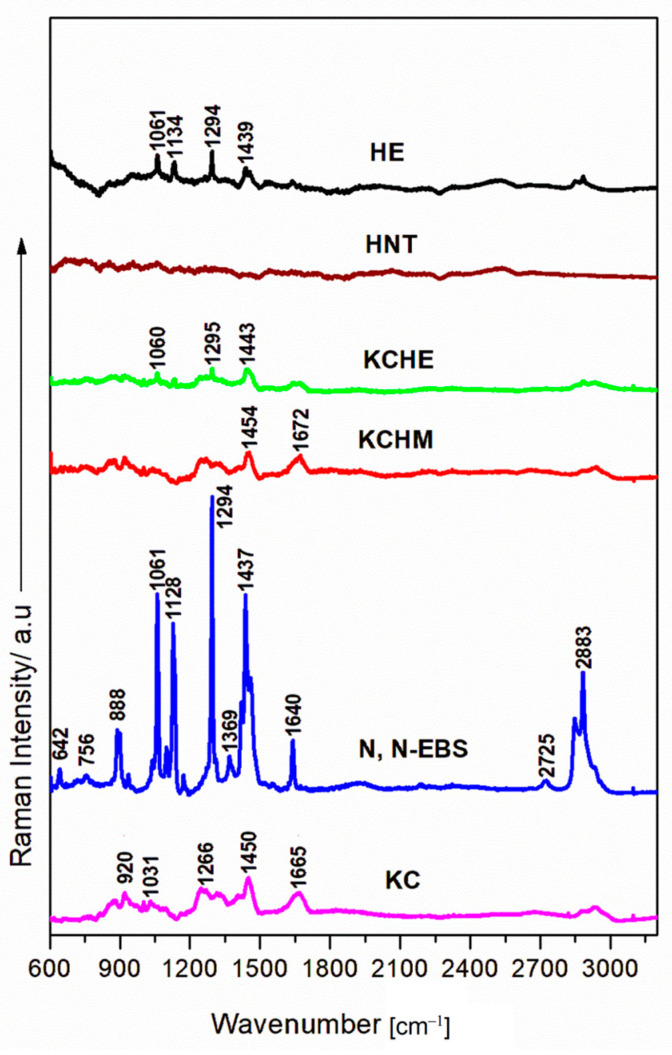
Raman spectra of KCHE and KCHM nanohybrids compared to KC, HNT, EBS, and HE nanoparticles powder.

Figure 4 shows the Raman spectra of KCHM and KCHE nanohybrids compared to those for HE, HNT, EBS, and KC powders. It can be seen that for the wavelength used (785 nm) for the analysis of these types of samples, the best-resolved ablation bands are of the EBS sample, which shows vibration modes characteristic for the highest peaks at 880 cm^−1^ CH_2_ rocking, 1061 cm^−1^ C-C stretch which is also found in HE and KCHE respectively, 1128 cm^−1^ C-C stretch, 1294 cm^−1^ CH_2_ twisting, 1437 cm^−1^ CH_2_ bending, 1640 cm^−1^ Amide I. Shifted bands around 1660 cm^−1^ can be observed in the case of HE and in the case of KCHM and KCHE but with a decreased intensity. In the case of HE, characteristic bands appear as in the case of EBS at: 1061, 1134, 1294, and 1493 cm^−1^. The intensity of the bands differs from EBS because, in HE powders, EBS is used in combination with HNT, which absorbs light radiation and has no Raman-specific fingerprint, as reported in the literature [35]. HNT is usually employed as an interlayer in complex material fabrication, being fully covered with other thin films to enhance their plasmonic properties, mainly for the topography of the surface (nanostructured morphology) [35,36]. In contrast to the FTIR fingerprint, HNT seems to have light Raman activity, showing no significant contribution in the visible up to IR laser excitation wavelengths. In the high wavenumbers region, EBS powder has the strongest peaks found at 2725 and 2883 cm^−1^ ascribed to the streching vibration of O-CH_3_. In line with FTIR analysis, EBS powder is the single compound that has significant spectral activity in this region.

#### 3.1.5. Thermal Analysis

- Thermogravimetric Analysis

**Figure 5:**
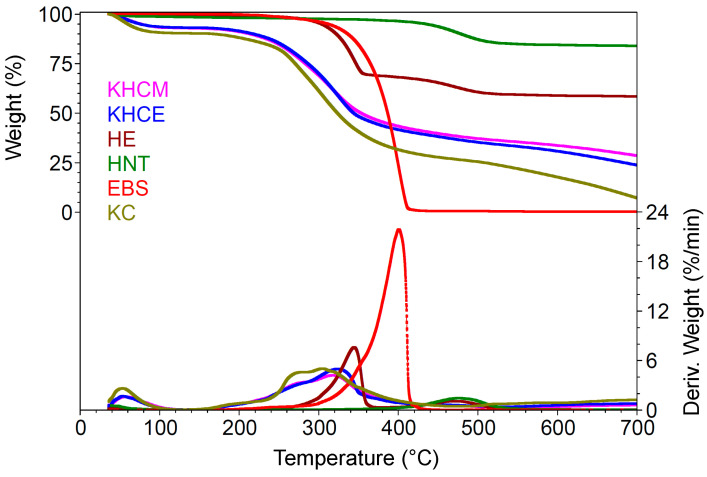
TGA and DTG curves for KCHM and KCHE nanohybrids compared to KC, HNT, EBS, and HE.

TGA and DTG results are shown in Figure 5 and Table 1. The hybrid containing halloysite with a surface modified with EBS (KCHE), apparently, has a slightly improved thermal stability compared to the hybrid containing unmodified halloysite (the temperature at the maximum rate of decomposition, Tmax, increases by almost 5 °C). However, if we consider the residue at 750 °C for each individual component and the percentage in which each component is in the composition of the hybrids, we can notice a difference between the calculated (theoretical) residue value and the practical one obtained (presented in Table 1). In the case of the KCHE hybrid, this difference is very small (approx. 0.2%), while for the KCHM hybrid, the difference between the theoretical value (21.97%) and the one in the table (25.52%) is 3.55%, which proves a better thermal stability, probably due to a better interaction between HNT and keratin. It can be seen that both hybrids have improved thermal stability compared to keratin (Tmax shifts to higher temperature by almost 10 °C). The thermal behavior of the two hybrids is similar to that of keratin and is characterized by three stages of decomposition. In the first stage of decomposition (RT-130 °C), the hybrids have a small weight loss of about 7%, which represents the removal of incorporated water. The second stage of decomposition (130–530 °C) is characterized by a higher percent of weight loss (57–59%) and represents the decomposition/denaturation of the keratin structure, which involves denaturation of the α-helix structure, the destruction of chain linkages, peptide bridges, and degradation of the skeletal. Mass loss (10–14%) in the last stage of decomposition (530–750 °C) signifies the decomposition of keratin into micromolecular products and volatile compounds (CO_2_, H_2_S, H_2_O, and HCN) [37,38].

- Differential Scanning Calorimetry Analysis

**Figure 6:**
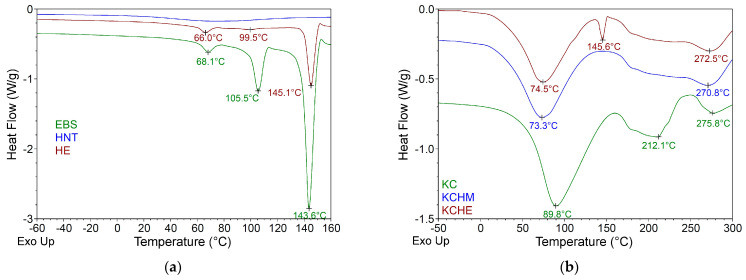
DSC curves for: (**a**) HNT, EBS, and HE; and (**b**) KCHM and KCHE nanohybrids compared to KC.

The DSC curves, recorded on the first heating cycle (Figure 6), reveal different endothermic peaks specific for each component. For EBS (Figure 6a), the three endothermic peaks at 68.1 °C, 105.5 °C, and 143.6 °C are assigned to the melting of α and β and the mixture of α and β crystalline forms of EBS [29,39]. For HNT modified with EBS (sample HE in Figure 6a), the endothermic peak at 105.5 °C decreased considerably and shifted to a temperature 10 °C lower (95.5 °C), while the other two peaks remained but shifted by 2.1 °C and 1.5 °C, respectively, toward lower temperatures, evidence of the interaction between HNT and EBS, preponderant in α crystalline form [22]. In the studied temperature range (−50–300 °C), keratin shows three endothermic peaks: at 89.9 °C, 212.1 °C, and 275.8 °C (Figure 6b). The first peak corresponds to the free water evaporation and coincides with the first stage of decomposition in TGA (Figure 5). The other endothermic peaks can be associated with the denaturation/destruction of the α helix structure and coincide with the second stage of decomposition in TGA (Figure 5). Brebu and Spiridon [12] studied the pyrolysis products and found that in the temperature range of 170–300 °C, NH_3_ and CO_2_ are formed at temperatures below 200 °C, and inorganic compounds with sulfur content are formed at temperatures above 240 °C. In the case of hybrids (Figure 6b), one can observe a disappearance of the endothermic peak at 212.1 °C and a shift of the other two endothermic peaks to lower temperatures (74.5 °C and 272.5 °C for KCHE, and 73.3 °C and 270.8 °C for KCHM), but with higher melting enthalpies at around 270 °C. In the case of the KCHE hybrid, an endothermic peak is observed at 145.6 °C, which represents the melting temperature of EBS. For hybrids, the temperature and the amount of heat required for water evaporation are lower. On the other hand, the amount of heat required for the degradation of the structure is about eight to nine times higher compared to keratin (ΔH is 16.5 J/g, 130.7 J/g, and 146.6 J/g for KC, KCHE, and KCHM, respectively) (Table 2). This behavior proves the existence of an interaction between HNT and keratin, which is stronger in the case of untreated HNT, as reflected in the improvement of the thermal stability of the hybrids. These results are in good agreement with the TGA results.

### 3.2. Characterization of Bio-Polyamide Nanocomposites

#### 3.2.1. SEM Analysis

**Figure 7:**
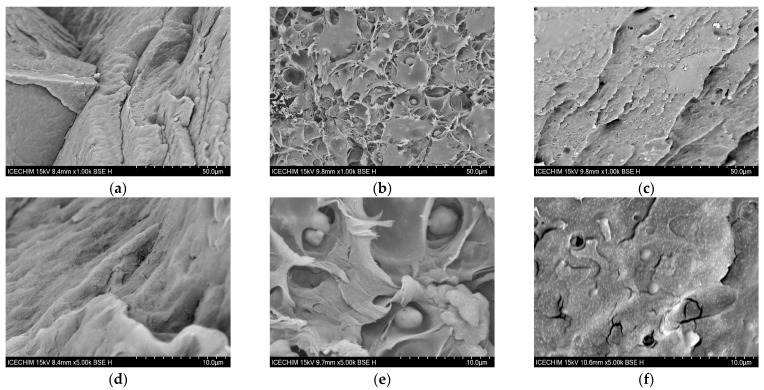
SEM images at ×1000 and ×5000 of fractured surfaces of PA–KCHM (**b**,**e**) and PA–KCHE (**c**,**f**) nanocomposites compared to PA (**a**,**d**).

The morphological characteristics of the fractured surface of PA and the PA–KCHM and PA–KCHE nanocomposites are shown in Figure 7. PA presents an irregular fracture surface with some roughness (Figure 7a,d), characteristic of ductile polymers [40]. The addition of the KCHM nanohybrid seems to produce a tear in the PA melt. KC particles modified with HNT are distributed uniformly in the polymer matrix in irregular gaps that they do not seem to interact with. It is observed that after the extrusion–injection processing, the KCHM particles are found in the form of small submicrons (<0.5 µm) or larger agglomerates (>2 µm; Figure 7b,e). In the case of PA–KCHE (Figure 7c,f), it is observed that the PA morphology is preserved. KCHE nanohybrid particles, generally submicron in size, are uniformly distributed and well embedded in the PA matrix. However, the presence of agglomerates can be observed, but of smaller size (1–2 µm) and fewer as in the case of PA–KCHM. This behavior is evidence of an interaction at the PA-nanohybrid interface. In Figure 7c,f, the micron and submicron holes left by the pull out of the dispersed particles from the PA matrix after fracturing are visible. The interruption of the continuity of the PA matrix and the appearance of tears in the case of PA–KCHM, as well as the existence of micro-holes in the case of PA–KCHE, we expect to be reflected in the decrease in the ductility of PA, by the decrease in elongation at break [41].

#### 3.2.2. X-ray Diffraction Analysis

**Figure 8:**
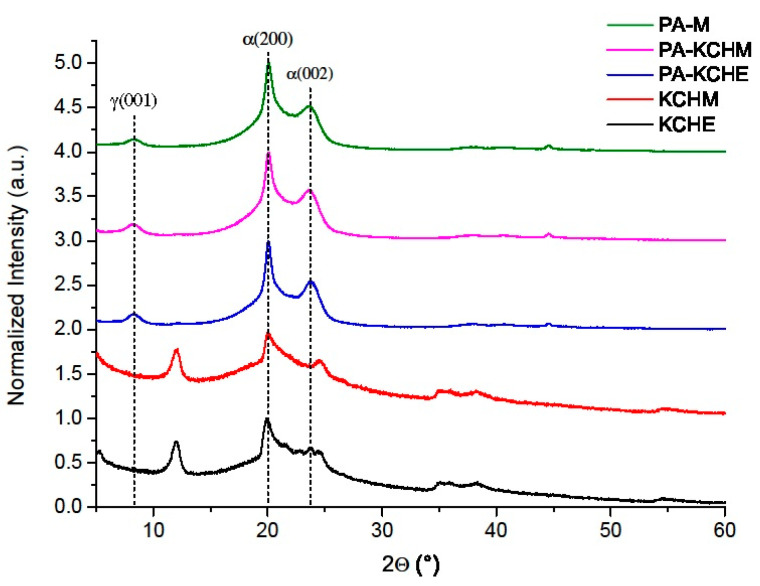
X-ray diffraction patterns of PA–KCHM and PA–KCHE nanocomposites compared to PA and nanohybrids.

The X-ray diffractograms of PA, PA–KCHM, and PA–KCHE nanocomposites are shown in Figure 8. The diffraction pattern of PA shows two main peaks at 2θ = 20.15° and 23.75°, corresponding to the α crystalline phase, the (200) and respectively (002) diffraction planes, and a small peak at 2θ = 8.24°, associated with the γ crystalline phase [42,43,44,45]. It has been shown that in the case of injection molding processing, PA6 and PA6-based nanocomposites crystallize differently along the thickness of the injected part, namely, in the skin, the proportion of the γ crystalline phase is preponderant, while the proportion of the crystalline phase grows toward the core of the injected part [46]. The addition of KCHM and KCHE nanohybrids did not change the crystalline structure of PA. However, it is observed that the FWHM and the height of the peaks change (Table 3). In the case of the peak corresponding to the γ(001) diffraction plane, an increase in intensity by 4% and 17% can be noticed for PA–KCHM and PA–KCHE, respectively. At the same time, the broadening of the peak and, respectively, its narrowing denote some disorder in the case of PA–KCHM (the crystallite size in γ(001) direction decreases by approx. 7%) and an ordering of the structure in the case of PA–KCHE (the crystallite size increases by approx. 3.6%). The peaks corresponding to the α crystalline form decrease in intensity, become narrower, and the crystallite size increases for both nanocomposites. The percent change of these peaks is higher for PA–KCHE (intensity decreases by 29–37% and crystallite size increases by 60–110%), which is evidence of a stronger interaction at the PA–KCHE nanohybrid interface. As a result, in the case of this nanocomposite, we can talk about an increase in the content of the α crystalline form, which should be reflected in the increase in the rigidity of the nanocomposite [47], taking into account that in the α form the interactions between the antiparallel macromolecular chains, through hydrogen bonds are stable and stronger than in γ form [48]. At the same time, we expect that the strength of this nanocomposite will not change as a result of maintaining and ordering the γ crystalline form.

#### 3.2.3. FTIR Analysis

**Figure 9:**
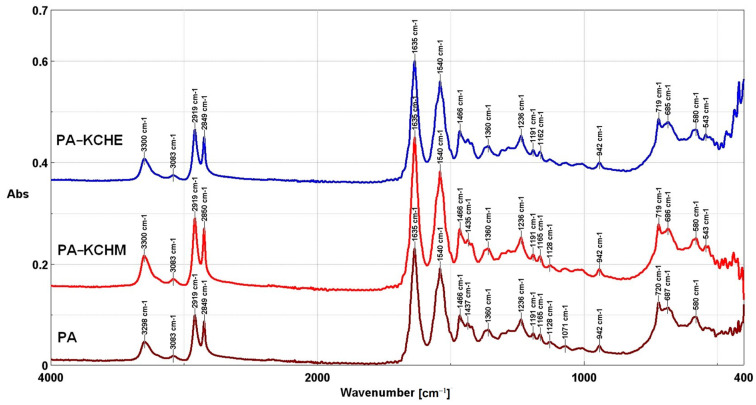
FTIR spectra of PA–KCHM and PA–KCHE nanocomposites compared to PA.

The FTIR spectra of PA nanocomposites compared to PA are presented in Figure 9. From the spectrum of PA, the bands at 942 cm^−1^, 1191 cm^−1^, and 1437 cm^−1^ can be identified, which is characteristic of CO-NH in a plane (α crystalline phase), C-CH bending (sym.) CH_2_ twisting (α crystalline phase,) and CH_2_ bending vibration next to a N-H group (γ crystalline phase) [49]. These results demonstrate that the crystalline structure of PA consists mainly of the α crystalline phase and a smaller percentage of the γ crystalline phase, in good agreement with the results of the XRD analysis. The bands associated with the amide groups at 3298 cm^−1^, 1635 cm^−1^, 1540 cm^−1^, and 1236 cm^−1^, similar to those identified for keratin, characteristic for Amide A, Amide I, Amide II, and Amide III, respectively, can also be identified. The peaks at 687 cm^−1^ and 580 cm^−1^, characteristic for –NH2 and –CHO groups, are specific only for PA [33]. New peaks are not observed in the FTIR spectra of the PA–KCHM and PA–KCHE nanocomposites because the peaks identified in the KCHM and KCHE nanohybrids (keratin and HNT-specific) overlap with those specific to PA. For this reason, it is difficult to identify variations in the intensity of the peaks, that could be evidence of physical interactions between the components.

#### 3.2.4. Raman Analysis

**Figure 10:**
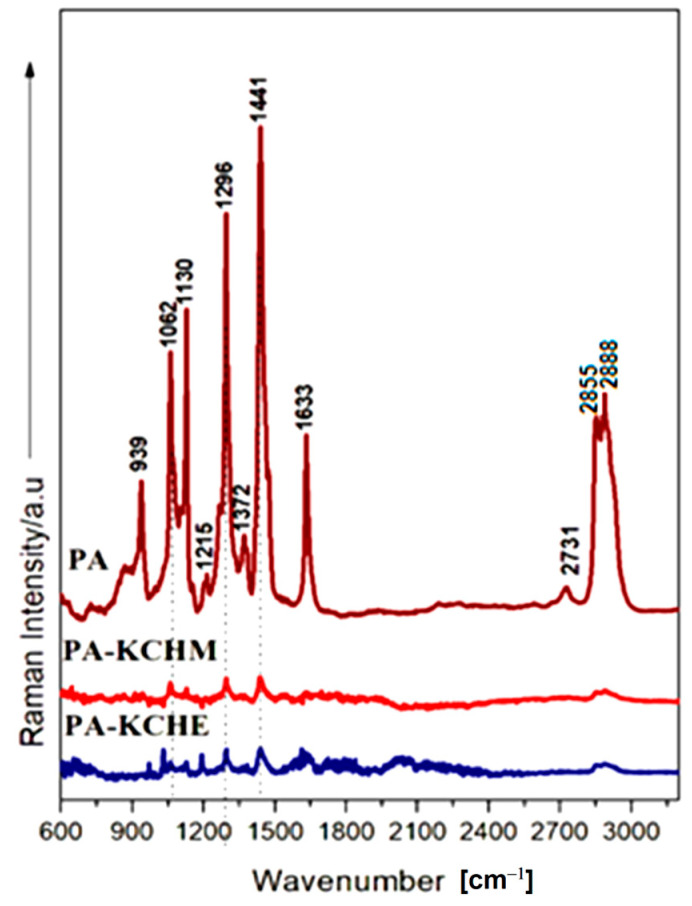
Raman spectra of PA–KCHM and PA–KCHE nanocomposites compared to PA.

Figure 10 depicts the Raman spectra of polyamide PA in comparison with those for nanocomposites PA–KCHM and PA–KCHE. The PA spectral profile contains characteristic polyamide bands found at 1633 cm^−1^ (Amide I) and 1296 cm^−1^ (Amide III), respectively, ascribed to the in-plane CN and C=O vibrations, associated with the amino group (-C(= O)-NHC-) bonded, unambiguously indicating the presence of the amide group in the polymer. Two intense bands are found at 2888 cm^−1^ and 2855 cm^−1^, belonging to symmetric and asymmetric CH_2_ stretching vibrations. Other bands present in the PA spectrum are found in the range 1400–850 cm^−1^ and belong to stretching and deformation vibrations of C-C bonds and CH_2_-rocking, as reported [50]. When comparing the Raman spectra of PA and PA–KCHM and PA–KCHE, a drastic decrease in the intensity of the characteristic polyamide bands was observed for nanocomposites. This spectral behavior is supported by the FTIR analysis, considering that no new IR active bands were revealed for the nanocomposites as compared to the PA-specific bands. We can assume that during the mixing process of PA with different types of nanohybrids, the materials that were practically used to fill the polymer matrix, the nanocomposites change their optical properties. By absorbing the laser light instead of scattering it, the Raman signal collected is reduced, and the specific Raman profile has lower intensities than a pure PA fingerprint.

#### 3.2.5. Thermal Analysis

- Thermogravimetric Analysis

**Figure 11:**
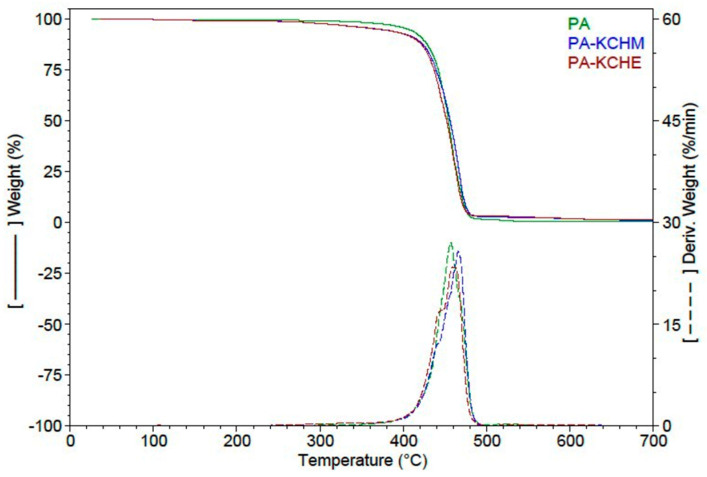
TGA and DTG curves of PA–KCHM and PA–KCHE nanocomposites compared to PA.

The thermal behavior of PA–KCHM and PA–KCHE nanocomposites compared to PA is shown in Figure 11, and the TGA results can be found in Table 4. From Figure 11, it can be seen that the nanocomposites show a thermal behavior similar to PA. From Table 4, it can be seen that up to 230 °C, the weight loss is below 1% and represents the removal of moisture as a result of the more or less hydrophilic nature of the components. In the temperature range 230–397 °C, the weight loss of the nanocomposites is higher than in the case of PA (of approx. 5.4% in the case of PA–KCHM and 6.4% in the case of PA–KCHE, compared to approx. 2.9% in the case of PA). In this temperature range, PA can lose the residual monomer and the fragments of the polymer chain [51], while in the case of nanocomposites, keratin, HNT, and EBS from nanohybrids start to decompose. The main stage of decomposition occurs in the temperature range 397–503 °C when the nanocomposites lose approx. 89% by weight with the maximum rate of decomposition at a temperature of 461–462 °C. In the last stage of decomposition, the weight loss is only 2–3% for the two nanocomposites, but the maximum decomposition rate moves toward temperatures of approx. 20 °C higher than PA. The PA–KCHE nanocomposite shows better thermal stability compared to PA–KCHM if we consider the lower weight loss (1.93% vs. 3.32%) and the higher residue at 700 °C (1.21% vs. 1.08%). Theoretically, the residue at 700 °C for PA–KCHM should have been by approx. 18% higher than for PA–KCHE, based on the composition and weight loss of each individual component. Practically, the residue at 700 °C for PA–KCHE is higher by approx. 11% compared to that for PA–KCHM, evidence of a stronger interaction between PA and the KCHE nanohybrid. These results are consistent with the XRD, FTIR, and SEM results.

- Differential Scanning Calorimetry Analysis

**Figure 12:**
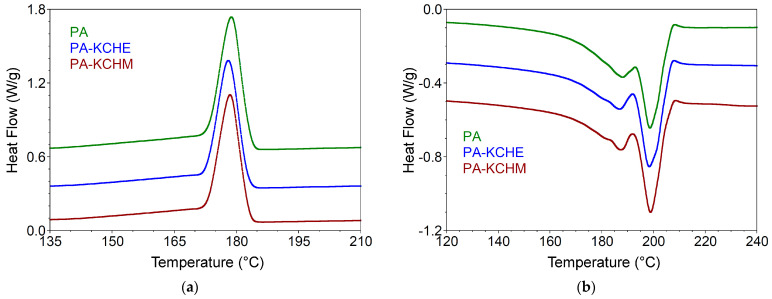
DSC curves for PA and the PA–KCHM and PA–KCHE nanocomposites: (**a**) first cooling; and (**b**) second heating.

Like Quiles-Carrillo et al. [6], the heating/cooling thermograms were represented in the temperature range of 120–240 °C to better highlight the melting/crystallization behavior of the samples. It can be seen from Figure 12a and Table 5 that PA, after the thermal history was removed during the first heating cycle, crystallized from the melt during cooling, showing a sharp exothermic peak at 178.8 °C (ΔH_c_ = 70.31 J/g). During reheating (the second heating cycle), two well-defined melting endotherms are observed at 188 °C (ΔH_m_ = 43.63 J/g) and 198.6 °C (ΔH_m_ = 27.96 J/g), respectively, which are due either to the existence of a mixed crystalline structure or to a melting-recrystallization process during reheating [52]. First, the γ crystalline form melts at 188 °C, which, through the recombination of the molecular chains, can recrystallize in the α crystalline form and melt at 198.6 °C [53]. The crystallinity for these forms is 17.9% and 11.5%, respectively, with the total crystallinity being 29.34%. For nanocomposites, the cold crystallization peak is practically the same, but the enthalpy of crystallization decreases by 6% for PA–KCHE and by 8% for PA–KCHM compared to PA. The decrease in crystallization enthalpy indicates changes in crystal formation. The number of crystallization nuclei decreases the most in the case of the PA–KCHM nanocomposite, but the crystallite growth is slower in the case of PA–KCHE (the crystallization temperature has the smallest value) [54]. The melting temperature of the γ crystallites (T_m1_) decreases by 1.2 °C in the case of PA–KCHE and by 0.6 °C in the case of PA–KCHM. The melting enthalpy, however, increases by 20% in the case of PA–KCHE and decreases by approx. 14% in the case of PA–KCHM. However, an increase in crystallinity (about 23% vs. about 16% for PA–KCHM) and the widest melting range (difference between melting temperature and onset) is observed for PA–KCHE. This behavior demonstrates that the melting of crystallites depends more on their size than on the degree of crystallinity. The larger the crystal size, the wider the melting range. According to the XRD results, the crystallite size for PA–KCHE is larger than that of PA–KCHM. In the case of melting α crystallites (T_m2_), a greater increase in crystallinity is observed in the case of PA–KCHE (by ca. 16% compared to ca. 11% in the case of PA–KCHM). These results prove a better interaction between the components in the case of PA–KCHE, which should be reflected in improved mechanical properties. These results are in good agreement with the XRD results.

#### 3.2.6. Mechanical Properties Analysis

**Figure 13:**
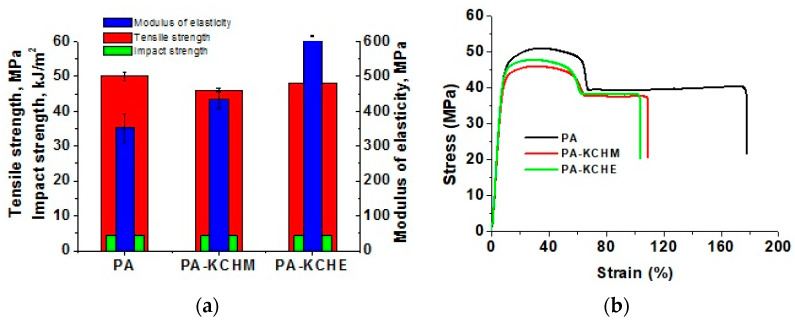
Mechanical properties of PA and the PA–KCHM and PA–KCHE nanocomposites: (**a**) tensile strength, modulus of elasticity, and impact strength; and (**b**) strain–stress curves.

The mechanical (tensile and impact) properties of PA and PA nanocomposites are presented in Figure 13. It can be observed that, compared to PA, in the case of nanocomposites, the maximum tensile strength (tensile stress at tensile strength) decreases slightly, by approx. 8% in the case of PA–KCHM and with approx. 4% in the case of PA–KCHE. The impact strength remains unchanged. Instead, the stiffness improves by approx. 24% in the case of PA–KCHM and with approx. 75% in the case of PA–KCHE (Figure 13a). From the stress–strain curves (Figure 13b), the ductile behavior of PA can be observed, namely the ability to deform plastically before breaking. Under an applied stress, the specimen initially undergoes an elastic, reversible deformation, and after reaching the tensile stress at yield, it begins to deform plastically (irreversibly); it necks and deforms further until it breaks. Fully bio-PA 1010 synthesized by Hernandez-Garcia et al. [7] showed almost the same behavior, with a tensile modulus of 762.1 ± 57.5 MPa, a yield strength of 45.4 ± 3.3 MPa and an elongation at break of 155.06 ± 36.3%, being characterized by the authors as a material with medium elasticity but high strength. Compared to this, the PA analyzed in this paper presented a larger range of plastic deformation and a lower stiffness, but a higher elongation at break. The nanocomposites keep their ductile character, but the elongation at break decreases (by approx. 39% in the case of PA–KCHM and by approx. 42% in the case of PA–KCHE), proving the decrease in chain mobility due to the interaction between the components. In general, the increase in the modulus of elasticity and the decrease in elongation at break are the result of the internal microstructure of PA and correlate with the increase in the degree of crystallinity [8]. These results are consistent with XRD and DSC and prove the reinforcing effect of the keratin–halloysite hybrid.

#### 3.2.7. Dynamic Mechanical Analysis (DMA)

**Figure 14:**
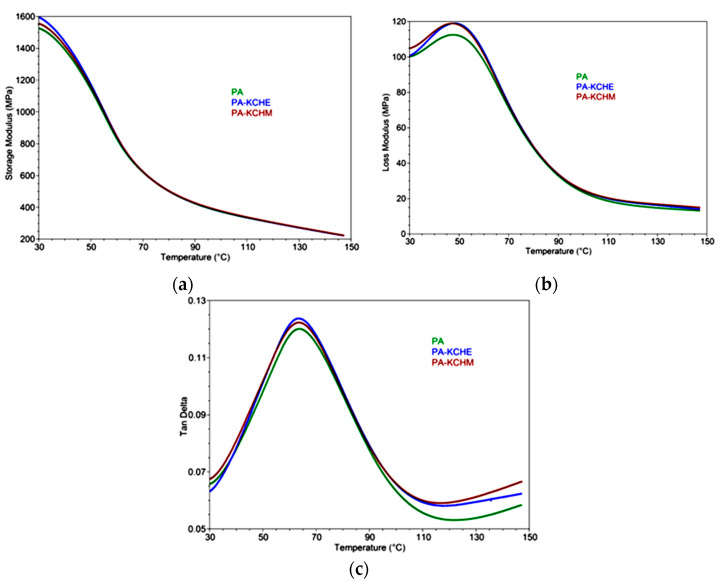
Storage modulus (**a**), loss modulus (**b**), and Tan Delta (**c**) vs. temperature for PA, PA–KCHM, and PA–KCHE nanocomposites.

Storage modulus (E′), loss modulus (E″), and loss factor (Tan δ) of PA, PA–KCHM and PA–KCHE nanocomposites are shown in Figure 14. Both the nanocomposites show a slightly increased E′ compared to PA on the whole range of tested temperatures (Figure 14a). Samples differentiate very well at temperatures below 60 °C, and PA–KCHE presents the highest storage modulus values. E′ at 30 °C is approx. 5% higher than that of PA. This increase in E′ is consistent with the increase in Young’s modulus of elasticity (Figure 13a). In fact, E′ at 30 °C shows the highest value for all samples, after which it decreases with increasing temperature. The E″ at 30 °C is almost similar for both nanocomposites but higher by approx. 5% than E″ of PA (Figure 14b). However, it is observed that E″ for PA–KCHE is slightly lower than for PA–KCHM, especially at 30 °C, proving a decrease in the mobility of the PA molecular chain due to a better interaction between PA and the KCHE nanohybrid [55]. From Table 6, it can be noticed the Tg value (from the tan δ vs. temperature plot) is almost the same for all samples (ca. 64 °C). According to some authors, the Tg of PA1010 is in the temperature range of 40–60 °C [40] or 60–80 °C [10] and is attributed to α relaxation. Table 6 also shows that the nanocomposites have higher values of the maximum peak of E″ (by approx. 6%) and that of tan δ (by approx. 3%) compared to PA. This behavior indicates an improvement of the viscous properties due to the good interaction between the components, which is reflected in good impact strength and thermal stability.

#### 3.2.8. Nanomechanical Properties Analysis

**Figure 15:**
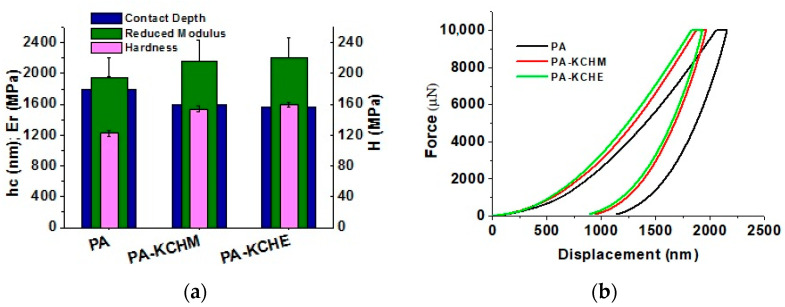
Nanomechanical properties of PA, PA–KCHM and PA–KCHE nanocomposites: (**a**) contact depth (hc), reduced modulus (Er), and hardness (H); (**b**) force vs. displacement curves.

The nanomechanical properties measured by nanoindentation are shown in Figure 15. It can be seen that the PA nanocomposites show an improved surface hardness and Er by approx. 26% and 11%, respectively, for PA–KCHM and with approx. 30% and 26%, respectively, for PA–KCHE. Surface hardness correlates very well with penetration depth; that is, the greater the indenter penetration depth, the lower the surface hardness. These values correlate with those obtained for the modulus of elasticity (Figure 13a) and the storage modulus (Figure 14a). The force vs. displacement curves (Figure 14b) confirm that PA–KCHE is the “hardest” sample, while PA is the “softest”. The depth of indentation at peak load, hmax, was 2154, 1962 and 1928 nm, while the final depth after unloading, hf, was 1063, 882 and 799 nm, for PA, PA–KCHM and respectively PA–KCHE. These results prove that PA–KCHE presents the highest degree of elastic recovery after unloading. This is also evidenced by the lowest value for hf/hmax ratio (0.49, 0.45, and 0.41 for PA, PA–KCHM, and PA–KCHE, respectively). According to Oliver, the lower limit (0 ≤ hf/hmax ≤ 1) corresponds to “fully elastic deformation” [56].

**Figure 16:**
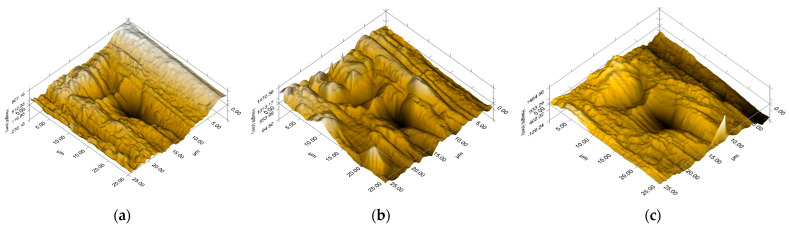
3D plot of SPM image after scratching for (**a**) PA; (**b**) PA–KCHM; and (**c**) PA–KCHE.

The scratching behavior of the nanocomposites compared to PA is highlighted by the 3D in situ topographic SPM images shown in Figure 16. Based on these images, the surface topography of the samples was characterized, and the surface roughness, friction coefficient, and scratch depth were calculated. The RMS roughness (root mean square roughness, R_q_), the coefficient of friction (μ), and the scratch depth (SD) are presented in Table 7. The results obtained revealed an improvement in scratch resistance in the case of nanocomposites. It can be noticed a decrease in the depth of the scratch by approx. 11% in the case of PA–KCHM and with approx. 17% in the case of PA–KCHE. These results are due to the presence of silicate nanoparticles, which are known to have a significant effect on scratch resistance [57]. At the same time, it is observed that PA–KCHE presents the lowest values for the coefficient of friction and surface roughness due to the presence in the composition of dispersion agent E, whose contribution to the improvement of resistance to friction and wear is also well known. These results are promising for use in the automotive industry, considering the importance of aesthetic properties in addition to the specific mechanical performance required by the field of application.

## 4. Conclusions

Mixing keratin (KC) with halloysite (H) under dynamic conditions led to obtaining a nanohybrid (KCHM) with improved thermal stability due to the high degree of interaction with KC, proven by SEM, XRD results, FTIR, RAMAN, TGA, and DSC.

Treatment of the H surface with ethylene bis stearamide (E) improved H dispersion by preventing the formation of agglomerates on the KC surface and maintaining its particle shape.

With the extrusion–injection processing process, the KCHE nanohybrid was uniformly distributed in the bio-PA matrix, and the degree of interaction at the interface with it was higher than in the case of the KCHM nanohybrid. This behavior, proven by the results of the SEM, XRD, FTIR, and DSC analyses, was reflected in the increase in PA crystallinity (i.e., the content in the alpha crystalline form and the size of the crystallites increased) and further in the increase in stiffness.

The results of thermal and (nano)mechanical analyses demonstrated that with only 5 wt.% KC and 1.43 wt.% H with the E-treated surface, bio-PA-based nanocomposites with thermal stability like PA at its processing temperature (210–230 °C), an improved modulus of elasticity of almost 75% (while maintaining impact and tensile strength), and improved aesthetic properties can be obtained. The hardness of the surface increased by approx. 30%, and scratch resistance improved (scratch depth approx. 17% lower and lower values for friction coefficient and surface roughness).

These results are proof that the new bio-PA nanocomposites can find applications in the automotive industry and are a promising alternative to synthetic polymer composites. Moreover, these new bio nanocomposites have the advantage that they contain non-abrasive natural fillers and can be processed into parts and recycled without problems compared to polymer composites based on glass fiber. Therefore, it is worth continuing the work and trying to enhance the properties of bio-PA with higher amounts of keratin, reducing the cost of bio-PA in this way.

## 5. Patents

A patent application on improving the properties of bio-PA using keratin/nanosilicate (or nanosilica) nanohybrids has been completed and registered with the State Office for Inventions and Trademarks.

## Figures and Tables

**Table 1:** TGA and DTG results for KCHM and KCHE nanohybrids compared to KC, HNT, EBS, and HE.

Sample	RT–130 °C	130–530 °C		530–750 °C	Residue at 750 °C
	Wt. Loss	Wt. Loss	Tmax	Wt. Loss	
	%	%	°C	%	%
EBS	0.00	99.69	400.4	0.15	0.16
HNT	1.34	13.29	476.9	1.27	84.10
KC	9.93	66.14	312.9	14.69	9.24
HE	0.55	39.97	344.2	1.26	58.22
KCHM	7.12	56.84	318.5	10.52	25.52
KCHE	6.80	59.26	323.3	14.05	19.89

**Table 2:** DSC results of KCHM and KCHE nanohybrids compared to KC, HNT, EBS, and HE.

First Heating	Enthalpy 1			Enthalpy 2			Enthalpy 3			Enthalpy 4			Enthalpy 5		
	Onset	Tmax	ΔH_m_	Onset	Tmax	ΔH_m_	Onset	Tmax	ΔH_m_	Onset	Tmax	ΔH_m_	Onset	Tmax	ΔH_m_
	°C	°C	J/(g)	°C	°C	J/(g)	°C	°C	J/(g)	°C	°C	J/(g)	°C	°C	J/(g)
EBS	60.8	68.1	7.0	99.6	105.5	33.4	138.6	143.6	122.9						
HE	59.2	66.0	4.5	75.8	99.5	3.3	139.9	145.1	33.7						
HNT	14.7	72.6	34.0												
KC	54.9	89.8	243.2							166.3	212.1	67.3	255.8	275.8	16.5
KCHE	35.6	74.5	160.1				140.0	145.6	5.8				162.7	272.5	130.7
KCHM	29.2	73.3	179.9										163.4	270.8	146.6

**Table 3:** XRD results for PA, PA–KCHM, and PA–KCHE.

Sample Name	PA Diffraction Plane	2θ (°)	d-Value (Å)	Height (cps)	FWHM (°)	Size (Å)
PA	γ(001)	8.24	10.73	7750	1.42	56
	α(200)	20.15	4.40	70,601	1.23	65
	α(002)	23.75	3.74	48,527	2.69	30
PA–KCHM	γ(001)	8.11	10.89	8096	1.52	52
	α(200)	20.09	4.42	53,922	1.17	69
	α(002)	23.63	3.76	28,429	2.23	36
PA–KCHE	γ(001)	8.77	10.67	9080	1.37	58
	α(200)	20.05	4.43	50,167	0.59	136
	α(002)	23.80	3.74	30,468	1.69	48

**Table 4:** TGA results of PA–KCHM and PA–KCHE nanocomposites compared to PA.

Sample	RT–230 °C	230–397 °C	397–503 °C		503–700 °C		Residue at 700 °C
	Wt. Loss	Wt. Loss	Wt. Loss	Tmax	Wt. Loss	Tmax	
	%	%	%	°C	%	°C	%
PA	0.56	2.93	94.86	465.2	1.28	531.2	0.37
PA–KCHM	0.89	5.36	89.35	462.3	3.32	550.7	1.08
PA–KCHE	0.90	6.41	89.55	461.3	1.93	552.0	1.21

**Table 5:** DSC first cooling and second heating results of PA–KCHM and PA–KCHE nanocomposites compared to PA.

Sample	Crystallization			Melting 1				Melting 2				Total	
	Onset	T_c_	ΔH_c_	Onset	T_m1_	ΔH_m1_	X_c1_	Onset	T_m2_	ΔH_m2_	X_c2_	ΔH_m_	X_c_
	°C	°C	J/(g)	°C	°C	J/(g)	%	°C	°C	J/(g)	%	J/(g)	%
PA	183.4	178.8	70.31	167.8	188.0	42.26	17.88	191.6	198.6	27.96	11.46	71.59	29.34
PA–KCHM	183.0	178.4	64.40	171.9	187.4	37.60	16.39	191.8	198.8	29.29	12.77	66.89	29.16
PA–KCHE	182.7	178.0	66.05	163.8	186.8	48.51	22.95	191.4	198.3	30.25	13.25	82.65	36.20

**Table 6:** DMA data of PA–KCHM and PA–KCHE nanocomposites compared to PA.

Sample	Step Transition-Storage Modulus			Loss Modulus, E″		Tan Delta	
	Onset	Midpoint (I)	End	Temperature	E″, Peak Max.	Temperature	Tan δ, Peak Max.
	°C	°C	°C	°C	MPa	°C	
PA	43.39	54.92	71.27	47.37	112.3	63.65	0.1200
PA–KCHE	42.75	54.69	70.82	47.83	118.8	63.76	0.1236
PA–KCHM	42.08	55.22	71.17	47.26	118.7	63.65	0.1222

**Table 7:** The RMS roughness, the coefficient of friction, and the scratch penetration depth measured for PA–KCHM and PA–KCHE nanocomposites compared to PA.

Sample	Rq (nm)	μ	SD (nm)
PA	146 ± 14	0.32 ± 0.007	463 ± 32
PA–KCHM	152 ± 9	0.33 ± 0.007	410 ± 40
PA–KCHE	137 ± 5	0.31 ± 0.001	383 ± 23

## Data Availability

The data presented in this study are available on request from the corresponding author.
